# Repeated Summer Drought and Re-watering during the First Growing Year of Oak (*Quercus petraea*) Delay Autumn Senescence and Bud Burst in the Following Spring

**DOI:** 10.3389/fpls.2016.00419

**Published:** 2016-03-31

**Authors:** Kristine Vander Mijnsbrugge, Arion Turcsán, Jorne Maes, Nils Duchêne, Steven Meeus, Kathy Steppe, Marijke Steenackers

**Affiliations:** ^1^Department of Forest Genetic Resources, Research Institute for Nature and ForestGeraardsbergen, Belgium; ^2^Department of Biometrics and Agricultural Informatics, Corvinus University of BudapestBudapest, Hungary; ^3^Department of Forest Reproductive Material and Plantation Management, Institute of Silviculture and Forest Protection, West-Hungarian UniversitySopron, Hungary; ^4^Department of Agro- and Biotechnology, School of Technology, Odisee University CollegeSint-Niklaas, Belgium; ^5^Laboratory of Plant Ecology, Faculty of Bioscience Engineering, Ghent UniversityGhent, Belgium

**Keywords:** drought, re-watering, oak provenance, seedling, survival, leaf senescence, bud burst, general linear mixed models

## Abstract

Climate change predicts harsher summer droughts for mid-latitudes in Europe. To enhance our understanding of the putative impacts on forest regeneration, we studied the response of oak seedlings (*Quercus petraea*) to water deficit. Potted seedlings originating from three locally sourced provenances were subjected to two successive drought periods during the first growing season each followed by a plentiful re-watering. Here, we describe survival and phenological responses after the second drought treatment, applying general linear mixed modeling. From the 441 drought treated seedlings 189 subsisted with higher chances of survival among smaller plants and among single plants per pot compared to doubles. Remarkably, survival was independent of the provenance, although relatively more plants had died off in two provenances compared to the third one with mean plant height being higher in one provenance and standard deviation of plant height being higher in the other. Timing of leaf senescence was clearly delayed after the severe drought treatment followed by re-watering, with two seedlings per pot showing a lesser retardation compared to single plants. This delay can be interpreted as a compensation time in which plants recover before entering the subsequent developmental process of leaf senescence, although it renders seedlings more vulnerable to early autumn frosts because of the delayed hardening of the shoots. Onset of bud flush in the subsequent spring still showed a significant but small delay in the drought treated group, independent of the number of seedlings per pot, and can be considered as an after effect of the delayed senescence. In both phenological models significant differences among the three provenances were detected independent from the treatment. The only provenance that is believed to be local of origin, displayed the earliest leaf senescence and the latest flushing, suggesting an adaptation to the local maritime climate. This provenance also displayed the highest standard deviation of plant height, which can be interpreted as an adaptation to variable and unpredictable weather conditions, favoring smaller plants in drought-prone summers and higher plants in more normal growing seasons.

## Introduction

Predicted climate change in temperate regions raises concerns about the ability of forest ecosystems to cope with longer and more severe summer drought periods. The intergovernmental panel on climate change (IPCC, 2014) expects not only a raise in mean temperature but also a higher frequency of extreme weather events. Forest vitality will be challenged and forests will become more vulnerable not only in Europe (Lindner et al., 2010), but all over the globe (Choat et al., 2012). For Europe, and more specifically for Belgium, climate projections predict increasing temperatures and irregular precipitation patterns in summer, augmenting the number and the intensity of drought periods (Baguis et al., 2010). A large part of the forests in the lower countries grows on sandy soils which are characterized by a relatively low water holding capacity, making them specifically vulnerable to extreme drought events during the growing season (Van der Werf et al., 2007), implying that drought and heat tolerance will become more critical for tree survival, especially considering additional stresses for trees by competition with others for light, water, and nutrient sources. Among important European tree species, oaks are well-known to be tolerant to drought (Leuschner et al., 2001), having a xeromorphic leaf structure and an adapted root structure that can cope with temporal and spatial variability in soil water and nutrient availability, and displaying an ability to rapidly resume assimilation after periods of water deficiency (Kubiske and Abrams, 1993; Galle et al., 2007; Kuster et al., 2013a,b). For this reason, oaks are put forward as promising candidate tree species to replace drought sensitive species such as beech (*Fagus sylvatica*) or spruce (*Picea abies*) on warm and dry sites in Europe (Leuschner et al., 2001). Apart from interspecific differences in degrees of drought tolerance, also among oak species, intraspecific variation may occur among provenances originating from varying growth sites displaying varying responses to drought. Provenances from xeric sites have been suggested to be better adapted to enhanced temperatures and lower water availability than provenances from more humid sites (Bruschi, 2010; Jensen and Hansen, 2010) although Arend et al. (2011) did not detect a correlation between climate at the site of origin and response to drought in different provenances of three European oak species. A reduced above-ground growth pattern with a diminished biomass production, together with a shift toward below-ground root growth, are well-established responses to drought in oak species (Broadmeadow and Jackson, 2000; Thomas and Gausling, 2000; Arend et al., 2011; Spiess et al., 2012; Kuster et al., 2013b) whereas the effects on phenology are less thoroughly examined and mainly described as an earlier stop of height growth under dry growing conditions (Jensen and Hansen, 2010; Spiess et al., 2012), also visible in an earlier cessation in secondary (radial) growth (Pflug et al., 2015) and which may show an after effect in the subsequent spring by an advanced bud burst (Kuster et al., 2014).

Plants have adapted the timing of their seasonal developmental processes to environmentally favorable periods of the year. Many aspects of the different phenophases that characterize growth in perennials, such as bud burst, growth cessation, senescence, bud set, and release from dormancy, are regulated by local climate (Rohde et al., 2011). In temperate regions, deciduous trees are prepared to the harshness of the winter by autumn senescing of the leaves, in which nutrients are efficiently remobilised before leaves are shed, and by hardening of the shoots to protect them against frost damage (Keskitalo et al., 2005). Autumn senescence in most trees is triggered by the photoperiod, a stable environmental cue that is considered to be more reliable than temperature as a harbinger of the first frosts (Keskitalo et al., 2005; Jackson, 2009). Still, concurrent with photoperiod, temperature has been shown to play a pivotal role in timing of the phenophases that determine the end of the growing season’s length in trees. Translocation of poplar clones (clonally replicated material) to different latitudinal growth sites revealed the effect of temperature, in conjunction with photoperiod, on timing and duration of the bud set process (Rohde et al., 2011). Apart from photoperiod and climatic factors, to which trees are evolutionary adapted, stress factors may influence the timing of growth stop, senescence and autumnal bud set. Plants may induce leaf senescence upon drought stress, allowing a reallocation of nutrients within the plant and thus enhancing its chances on survival, an adaptive mechanism that is well-studied among Mediterranean plant species (Munne-Bosch and Alegre, 2004).

The seedling stage of a forest tree is known to be the most vulnerable phase in its life cycle and therefore, understanding the stress responses of seedlings is crucial for predicting forest tree growth and survival (Niinemets, 2010; Psidova et al., 2015). Oak forests in Belgium are mainly small and fragmented, and additionally originated largely from plantations with forest reproductive material, although in recent decades natural rejuvenation has been emphasized and promoted for forest regeneration. In the context of climate change, information regarding sessile oak provenances tolerant to drought becomes more important, particularly for forest management and future (re)forestation. The purpose of this paper is to examine the impact of severe summer droughts on the survival of first year seedlings of three different sessile oak provenances, sourced in the same region in Belgium but with a variable origin, and on the phenology of the surviving seedlings in this vulnerable phase of their life. We specifically aimed to determine the survival rate and the effect on leaf senescence in the first growing season and on bud burst in the subsequent spring for surviving seedlings, all in relation to the origin and the size of the seedlings, the degree of water deficit and the number of seedlings per pot. We tested the hypothesis that sessile oak which is believed to be local of origin is more drought–tolerant compared to putative non-local origins.

## Materials and Methods

### Source Material

**FIGURE 1:**
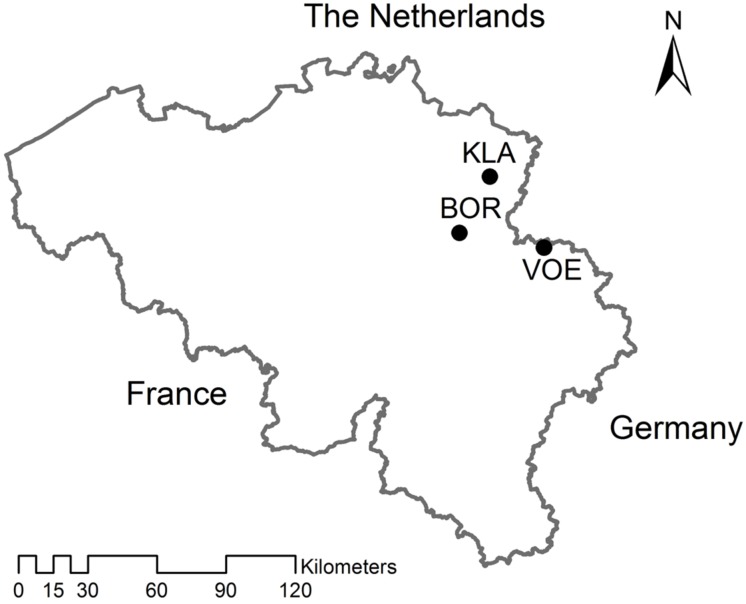
**Map showing the three sampled *Quercus petraea* stands in Belgium.** KLA: Klaverberg, VOE: Voeren, BOR: Borgloon.

Three provenances of *Quercus petraea* were chosen in Flanders, the northern part of Belgium, with deviating stand structure and history: Klaverberg (KLA), Voeren (VOE), and Borgloon (BOR; **Figure 1**). Acorns were collected per mother tree at the end of October 2013. KLA is a small relict of abandoned oak coppice wood growing on inland sand dunes within a former heath land. The oaks here are most probably local of origin (Vander Mijnsbrugge et al., 2003). Coppice wood in the former heath land was exploited by local poor farmers that would not buy planting stock when local sources were available. Acorns were collected from 13 visually older abandoned coppice stools. As the oaks are mostly growing widely spaced on the sand dunes, the chance on mixing acorns from different mother trees was negligible. VOE is a classical planted forest stand, even aged and approximately 80 years-old, growing on a loamy soil type. As is the case for the vast majority of such planted oak stands in Flanders, the origin of the original planting stock is unknown. BOR is a similarly planted forest and approximately 100 years-old, of which the origin of the planted material is also unknown. Here, the oaks grow on sandy soil. Acorns were collected underneath 14 dominant trees in VOE and three dominant trees in BOR, which all showed a well-developed crown. Collection was performed only close to the stem, minimizing the chance on mixing acorns between different mother trees.

### Germination of the Acorns

In November 2013, the collected seeds were sown in forestry trays with two seeds per cell, using standard nursery potting soil. During winter, the trays were watered manually keeping the soil moist. The experiment was located in a greenhouse with automatic temperature regulation, keeping the greenhouse frost-free in wintertime, but without additional heating. An automatic internal gray shade cloth system operates in the greenhouse, protecting the plants from high levels of insulation. In total, 1015 seeds germinated, 486 from KLA, 431 from VOE and 145 from BOR. All germinating plants were given water at regular times according to the visual needs as judged by experienced greenhouse workers. Seedlings were transferred in April 2014 to 1-lt pots (12 cm × 11 cm × 11 cm) using standard nursery potting soil [organic matter 20%, pH 5.0–6.5, Electrical Conductivity (EC): 450 μS/cm, dry matter 25%, fertilization: 1.5 kg/m^3^ powdered compound fertilizer NPK 12 + 14 + 24]. As not all seeds had germinated, both cells with only one seedling and cells with two seedlings were present. While transferring the seedlings, double plants in one tray cell were kept together. After the transfer, the seedlings were not additionally fertilized. For our experiment we choose to use seedlings in pots, rather than working in a field experiment outdoor, as this allowed to impose a drought period on a subset of plants while both treated and control plants could be subjected to very similar other growth conditions (light, temperature, nutrient availability, …). Furthermore, it allowed monitoring the reduction in water availability by weighing of the individual pots.

### Drought Treatment, Measurements, and Scoring

**FIGURE 2:**
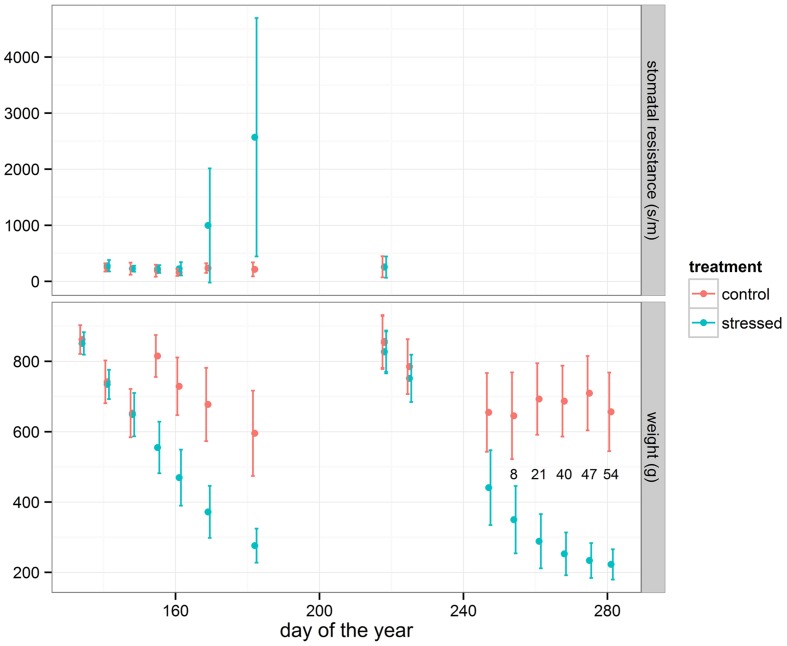
**Average and standard deviation of weight loss of the pots in the control and the stressed group of plants during the two drought periods.** Corresponding average and standard deviation of stomatal resistance (subset of 30 plants for each treatment) during the first treatment and relative amount (in %) of seedlings displaying visual stress symptoms (subset of 100 plants for drought treated group) during the second treatment are shown.

The pots were divided in two groups: a control and a treatment group. In both groups the three provenances were individually mingled at random (completely randomized). On 15th May and 6th August 2014 respectively the two groups of plants were soaked overnight to a fully water saturated condition in a basin with the water level up to two cm above the bottom of the pots. Up to 1st July and 17th October 2014 respectively the drought-treated group was not watered anymore, whereas the control group was further watered according to the visual needs of the plants. All plants were re-watered on 2nd July and 18th October 2014 respectively by soaking the two groups of plants in the same basin in the same way. After these drought periods and the re-watering, both groups were kept in well-watered conditions according to the visual needs of the plants. The first drought period lasted until stress was detected in stomatal conductance as measured by a porometer on a subset of plants (**Figure 2**). For this, 30 pots with relative high plants were randomly chosen from the control group as well as 30 from the drought-treated group to monitor the treatment effect. Leaf stomatal aperture in terms of leaf resistance to water vapor was measured weekly with a diffusion porometer (Model AP4, Delta-T Devices, Cambridge, UK) during the entire first drought period. As stomata are sensitive to drought stress, high resistance values represent a closing reaction and declining stomatal conductance and leaf assimilation rate. The porometer measurements were conducted during daytime between 10 a.m. and 3 p.m. In the period directly following re-watering after the first drought period, an extra growth flush was detected mainly among the stressed plants (Turcsán et al., 2016). The second drought period lasted until a large amount of plants showed visual signs of stress (wilting and/or curling of the leaves) and started dying off. During this second drought period, the wilting and/or curling of the leaves in the treatment group of plants was monitored visually on a weekly basis on 100 random plants. The number of plants with clear visual stress symptoms was counted, as indicated in **Figure 2**. The data of the second drought period are presented here. Therefore, reference to a drought period in this paper concerns the second drought period. After the second re-watering, all plants were kept well-watered according to visual needs, also during the winter and the following spring.

During the drought period all pots were weighed nearly weekly to measure the water loss (**Figure 2**). The initial weight at the beginning of the treatment period was measured after the pots had drained the excess of water. At the end of the treatment period drought symptoms had become clearly visible and plants had started dying off (**Figure 2**). As an approximation for the level of water deficit experienced by the seedlings, the weight loss of the individual pots at the end of the drought period, just before re-watering, was calculated relative to the initial weight at the beginning of the treatment: relative weight loss = (weight doy 218 – weight doy 281)/weight doy 218. This approximation is related to the soil relative water content (SRWC) which is the ratio between present soil moisture and field capacity (Xu et al., 2009), and is applicable to a large amount of potted plants.

During the drought period a large amount of plants died off. Therefore, survival was monitored as a separate binary variable. The height of the seedlings was measured with a ruler at the end of the first drought treatment (on 1st July 2014), at full recovery of the plants (on 4th September 2014) and at the end of the growing season (on 1st of December 2014). Two phenophases were scored on the plants: leaf senescence in autumn 2014 and bud burst in 2015. Leaf senescence on the surviving plants after the second drought treatment and the control group was scored following a eight level scoring protocol (**Table 1**) on 1st and 8th December (doy 335 and 343). Here, all the leaves of a seedlings were observed together and a visual mean of color change was made. Bud burst and leaf unfolding in the apical bud in the following spring was scored according to a six levels scoring protocol (**Table 1**) on 22th and 30th April, and 8th May (doy 112, 120 and 128).

**Table 1:** Description of the score levels of the two phenological response variables leaf senescence and bud burst in oak seedlings (*Quercus petraea*).

Phenophase	Score level	Description
Leaf senescence	1	Normal dark green leaves
	2	Light green leaves
	3	Light green leaves with yellow parts
	4	Yellow leaves still having green parts
	5	Yellow leaves with brown parts
	6	Brown leaves still having yellow parts
	7	Brown leaves
	8	Leaves shed

Bud burst	1	Apical bud in winter rest
	2	Apical bud swollen
	3	Apical bud opening, leaves not yet protruding
	4	Leaves protruding but not yet unfolding
	5	Leaves unfolding but not yet fully unfolded
	6	Leaves fully unfolded


### Data Analysis

All statistical analyses were performed in the open source software R 3.1.2 (R Core Team, 2014). Three response variables were modeled using generalized linear mixed models: survival, leaf senescence and bud burst. The number of seedlings used in each model are indicated in **Table 2**. Survival was examined using logistic regression (generalized linear mixed models) in the package lme4 (Bates et al., 2015), whereas the phenological response variables were modeled using cumulative logistic regression in the package ordinal (Christensen, 2013). The command clmm in the package ordinal models the chance to maximally have reached a given level of the ordinal response variable. We ordered the score levels of leaf senescence and bud burst in decreasing order, so that the chance to have maximally reached, e.g., leaf senescence score 4 equalled the chance to have reached maximally score 4, which included scores 8, 7, 6, 5 and 4, which can be interpreted as having reached at least score 4.

**Table 2:** Number of oak seedlings (*Q. petraea*) in this study (n) for the different modeled response variables survival (n_su_), leaf senescence (n_ls_) and bud burst (n_bb_), and according to treatment, provenance and number of seedlings per pot.

Treatment	Provenance	Seedlings per pot	n_su_	n_a_ (n_a_%)	n_ls_	n_bb_
Control	KLA	1	65	65 (100)	43	65
		2	162	155 (96)	104	155
	VOE	1	52	52 (100)	40	52
		2	125	121 (97)	96	121
	BOR	1	11	11 (100)	10	11
		2	46	45 (98)	45	45

Stressed	KLA	1	50	30 (60)	19	30
		2	167	51 (31)	30	51
	VOE	1	55	37 (67)	24	37
		2	107	56 (52)	29	56
	BOR	1	10	6 (60)	5	6
		2	52	9 (17)	7	9


*For the response variable survival, the number of surviving plants are indicated (n_a_), also expressed in % (n_a_%).*

In the fixed part of the models several covariates were examined for significant explanatory power: the plant height before the second drought treatment (continuous variable), the provenance of the seedlings (factor variable) and the number of seedlings per pot (factor variable). As the plants did not show a height increment during or after the second drought period, the plant height before the second drought period was the final height growth of the growing season 2014. The two phenological models leaf senescence and bud burst got an additional covariate, day of observation, as for these response variables repeated observations per plant were available. All three covariates height, provenance and seedlings per pot were first included in each of the three models, survival, leaf senescence and bud burst, with an interaction term with weight loss of the pots relative to the fully water saturated condition during the second drought period. In this way the influence of the drought treatment was examined. In all models the mother plant from which acorns were collected was in the random part (random intercept). For the phenological models, an additional unique plant identity variable was added in the random part of the models (random intercept) to account for the repeated measurements on the same plants. Using drop 1 (a likelihood ratio test) the fixed part of all three models was reduced up to only significant terms. With a significant interaction term, the corresponding covariates (main effects) remained in the model.

The chance (p) of survival was calculated following a logistic regression:

$$\log(p/\left(1-p\right))=\alpha +\beta_{P}P+\beta_{H}H+\beta_{S}S+\beta_{W}W+\beta_{P}PW+\beta_{H}HW+\beta_{S}SW$$

with α as the estimated intercept and the β’s as the estimated parameters of the fitted model. Shown here is the full model with all covariates and interaction terms. P is the provenance (KLA, VOE, and BOR), W is the relative weight loss of the pots accounting for the stressed condition, H is the plant height and S is the number of seedlings per pot (1 or 2). The model was reduced up to only significant terms.

For leaf senescence and bud burst, the chance (p) to have reached at least a given phenological score level on a given day was calculated following a cumulative logistic regression:

$$\log(p/\left(1-p\right))=\alpha_{T}-\beta_{D}D-\beta_{P}P-\beta_{H}H-\beta_{S}S-\beta_{W}W-\beta_{P}PW-\beta_{H}HW-\beta_{S}SW$$

where the β’s are the estimated parameters of the fitted model and with α_T_ as an estimated threshold value for the passing on from one level of the phenological variable to the next. D is the day of observation. Based on the formulas of the reduced models with only significant terms in the fixed part, time lags were calculated between control and stressed condition, or between the different provenances or between single and double plants per pot.

## Results

### Survival

**FIGURE 3:**
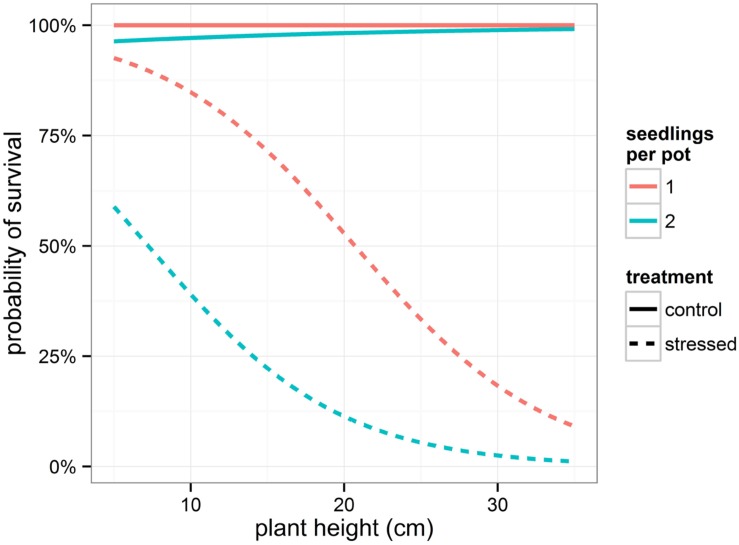
**Modeled probability of survival depending on treatment and on number of seedlings per pot.** To calculate the probabilities, the mean relative weight loss for the control and stressed group of plants was applied.

The binary response variable survival indicating whether or not a seedling survived the drought period, was modeled using generalized linear mixed models. Influencing factors were the height of the seedlings and the number of seedlings per pot, both depending on the amount of relative weight loss (significant interaction terms, **Table 3**). Interestingly, provenance was not significant in this model, indicating that the provenance of the seedlings did not affect survival rate. Seedlings that shared a pot displayed a lower probability of survival in the drought stressed condition (**Table 3**; **Figure 3**). In addition, the higher the plants, the greater the probability to die off in the stressed condition (**Table 3**; **Figure 3**).

**Table 3:** Model statistics for the general linear mixed model of the binary response variable survival.

Covariate	Estimate	*SE*	*z*-value	*P*-value
Intercept	56.2	15.7	3.57	**<0.001**^∗∗∗^
W	-0.72	0.21	-3.42	**<0.001**^∗∗∗^
H	0.15	0.08	1.82	0.068
S	-52.3	15.7	-3.34	**<0.001**^∗∗∗^
H:W	0.0042	0.0012	-3.38	**<0.001**^∗∗∗^
S:W	0.68	0.21	3.26	**0.001**^∗∗^


*W: relative weight loss (continuous variable), H: height of the plant (continuous variable), S: number of seedlings per pot (factor variable with single plant per pot as standard level). Significant results are in bold: ^∗∗^*P* < 0.01; ^∗∗∗^*P* < 0.001.*

### Leaf Senescence

**FIGURE 4:**
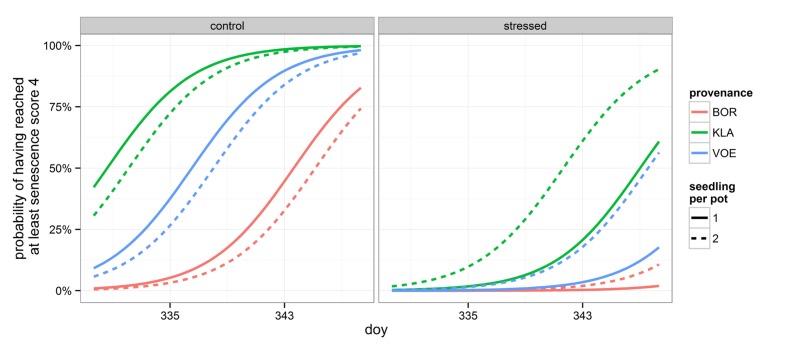
**Modeled probability of having reached at least leaf senescence score 4 (yellowing leaves) for the control and the stressed group of plants, depending on the provenance and on the number of seedlings per pot.** To calculate the probabilities, the mean relative weight loss of the pots for the control and stressed group of plants was applied. doy: day of the year.

**FIGURE 5:**
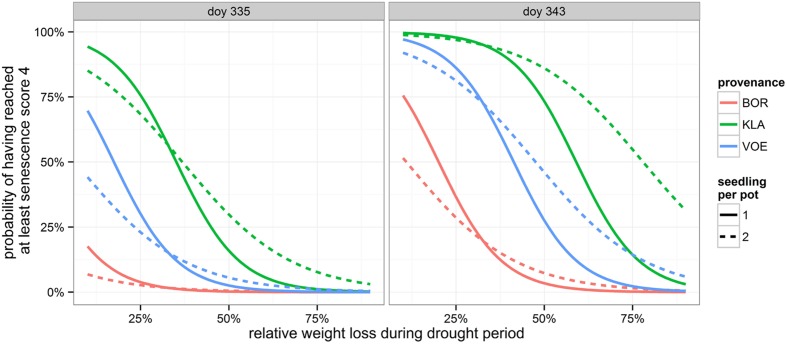
**Modeled probability of having reached at least leaf senescence score 4 (yellowing leaves) on the two observation days, depending on the provenance and on the number of seedlings per pot.** The higher the relative weight loss, the higher the drought stress. doy: day of the year.

Decolouration and finally shedding of the leaves was scored in the autumn of 2014, following a severe drought and re-watering period. In the phenological model, provenance (without interaction term) and the interaction between number of seedlings per pot and relative weight loss appeared significant (**Table 4**). With a more severe drought stress, as expressed by a high relative weight loss, decolouration of the leaves was clearly retarded (**Figures 4** and **5**). Both in the control group as in the drought treated group (thus independent of the drought treatment), leaf senescence appeared first in KLA, than in VOE and finally in BOR (**Figures 4** and **5**). The significant interaction term with seedling per pot was visualized by differing steepness of the modeled S curves between single and double plants per pot (**Figure 5**) indicating that among the double plants per pot in stressed conditions senescence of the leaves was less retarded compared to single plants. According to the fitted model, the control and stressed group of plants differed 18.8 days in timing of leaf senescence for single plants per pot. In the control group double plants per pot senesced 1.5 days later compared to the single plants, whereas they senesced 6.4 days earlier than the singles in the stressed condition. For the different provenances, a time lag was observed between KLA and VOE of 6.7 days and between KLA and BOR of 14.7 days (independent of the drought treatment).

**Table 4:** Model statistics for the general linear mixed model of the ordinal phenological response variables.

	Leaf senescence				Bud burst			
		
Covariate	Estimate	*SE*	*z*-value	*P*-value	Estimate	*SE*	*z*-value	*P*-value
D	-0.30	0.021	-14.1	**<0.001**^∗∗∗^	-1.039	0.043	-24.35	**<0.001**^∗∗∗^
VOE	1.98	0.68	2.90	**0.004**^∗∗^	-4.177	0.757	-5.52	**<0.001**^∗∗∗^
BOR	4.35	1.01	4.29	**<0.001**^∗∗∗^	-3.120	1.201	-2.60	**0.009**^∗∗^
W	0.11	0.016	7.07	**<0.001**^∗∗∗^	0.032	0.007	4.36	**<0.001**^∗∗∗^
S	1.54	0.80	1.91	0.056				
S:W	-0.047	0.019	-2.53	**0.011**^∗^				
H					0.187	0.036	5.22	**<0.001**^∗∗∗^


*D: day of observation (continuous variable), VOE and BOR: oak provenances that are compared to the standard provenance KLA (factor variable), W: relative weight loss (continuous variable), S: number of seedlings per pot (factor variable with single plant per pot as standard level), H: height of the plant (continuous variable).*

*Significant results are in bold: ^∗^*P* < 0.05; ^∗∗^*P* < 0.01; ^∗∗∗^*P* < 0.001.*

### Bud Burst

**FIGURE 6:**
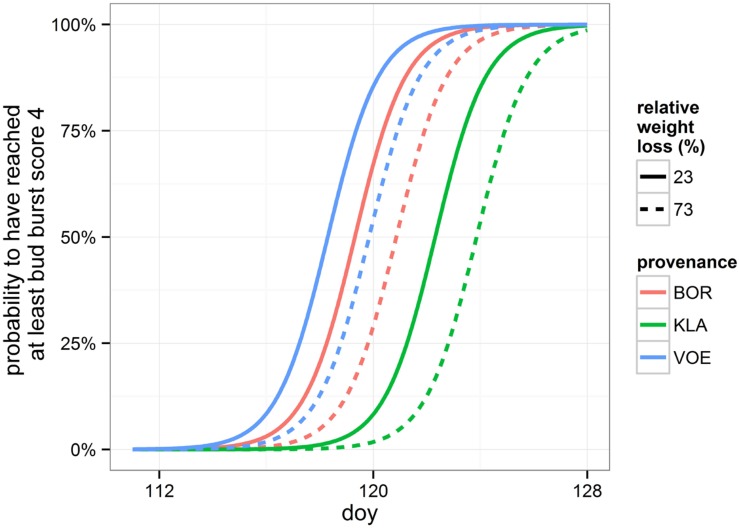
**Modeled probability of having reached at least leaf bud burst score 4 (leaves protruding from the bud), depending on the provenance and on the relative weight loss.** To calculate the probabilities, the mean relative weight loss of the pots for the control (23%) and stressed group of plants (73%) was applied. doy: day of the year.

**FIGURE 7:**
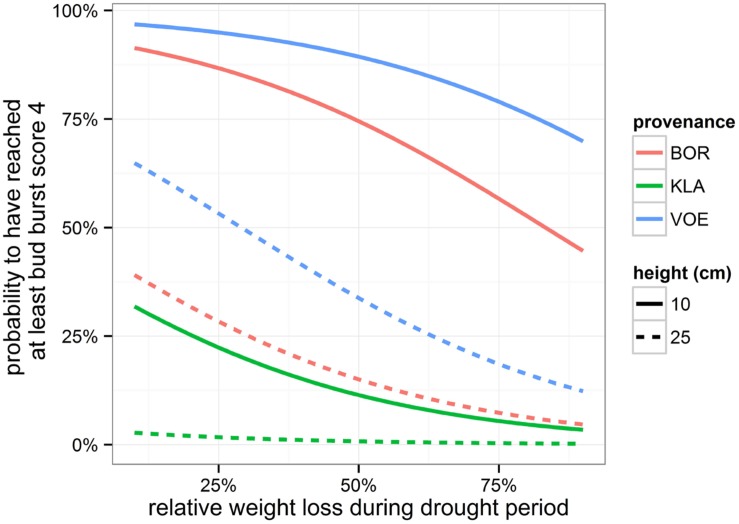
**Modeled probability of having reached at least bud burst score 4 (leaves protruding from the bud), depending on the provenance and on the height of the plants.** Probabilities are calculated for 1st May. The higher the relative weight loss, the higher the drought stress.

Bursting of the buds and leaf unfolding was observed in the spring following the growing season with drought treatments. Modeling this ordinal phenological variable revealed a significant influence of relative weight loss, indicating that the drought treated group of plants burst buds later compared to the control group (**Table 4**; **Figure 6**). Provenance and plant height were covariates in the model with a significant explanatory power, both independent of the treatment (no significant interaction terms with relative weight loss). The difference in timing of bud burst between the control and stressed group of plants was 1.5 days. The provenances VOE and BOR flushed earlier than KLA (**Figure 6** with VOE and KLA differing 4 days) and smaller plants burst buds earlier than larger plants (**Figure 7**), both independent of the relative weight loss of the pots (no significant interaction term with relative weight loss).

## Discussion

As climate change is predicted to augment the length and severity of drought periods during the growing season in Belgium, we studied the impact of water deficit in the first year on survival of three provenances of sessile oak seedlings (*Q. petraea*), and on the phenology of surviving plants. Our central aim was to contribute to a better understanding of possible impacts of summer droughts on forest regeneration. Two major results can be stressed. Firstly, there was no significant difference in survival after a severe summer drought period between the three local provenances. Secondly, the drought period followed by a plentiful re-watering induced a retarded leaf senescence with a delayed bud burst, likely as an after affect, in the subsequent spring.

### Impact of Provenance

**FIGURE 8:**
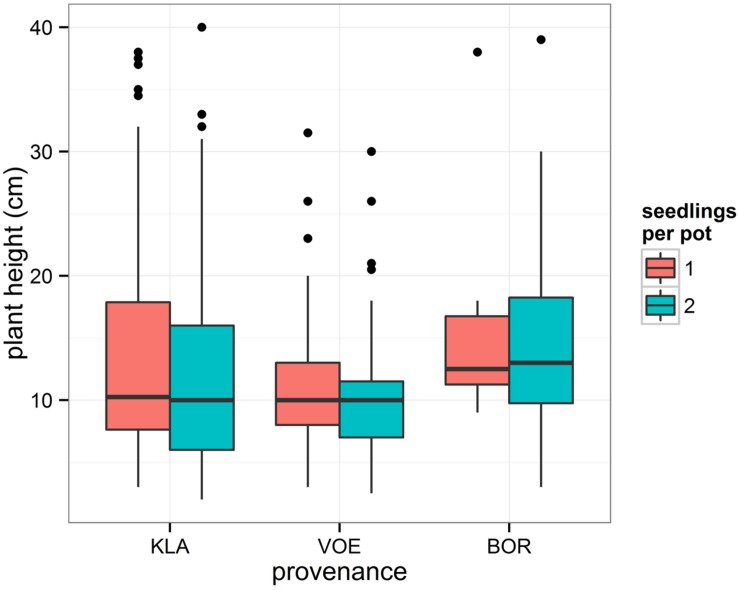
**Boxplot representing the height of the seedlings in the drought stressed group according to the provenance and the number of seedlings per pot.** Number of plants in each box are in **Table 2** (n_su_ of stressed group).

Survival of seedlings after severe water deficit appeared independent of the provenance. Still, a higher number of plants of KLA and BOR had died off compared to VOE (**Table 2**), because the model accounted for the height of the plants with smaller plants having a higher chance of survival. BOR was characterized by a higher mean height of plants and KLA did not show a higher mean height but a higher standard deviation compared to VOE, implying a relative larger number of high plants being more prone to starvation by drought (**Figure 8**). KLA is believed to be local of origin (Vander Mijnsbrugge et al., 2005). A cpDNA analysis revealed a uniform haplotype that fits in the reconstructed postglacial migration routes (Vander Mijnsbrugge et al., 2003). In addition, the mother trees are abandoned remnants of coppice wood in former heath land where no tradition existed among the relatively poor farmers of introducing foreign provenances. In a provenance trial, located in Belgium, KLA flushes later compared to several commercial provenances from western Germany (data not shown) indicating an adaptation to the less predictable weather (maritime climate) caused by oceanic influences with a smaller contrast between summer and winter and less predictable transitions. Other Belgian commercial provenances in this trial are typical planted forest stands, comparable to VOE and BOR, displaying an earlier flushing in spring, likely indicating a non-local origin of the mother trees. Similarly, in the here presented experiment leaves senesce earlier in KLA compared to VOE and BOR and, in addition, buds flush later, most probably indicating a shorter growing season as an adaptation to a more unpredictable temperate maritime climate for KLA and a non-local origin for VOE and BOR. The latter is likely as forest history in the northern part of Belgium is characterized by successive phases of deforestation and afforestation in this densely populated region (Vandekerkhove et al., 2011) resulting in a scattered and fragmented landscape, and in mostly relatively small oak forests of which a vast majority has a plantation origin, largely with an unknown origin of the planting stock. German origins of planting stock may have been planted as war reparations after the two world wars. The result stresses the importance of local provenances such as KLA not only because of the local adaptation to current climate as expressed by the phenological responses, but also because of putative larger variability in quantitative traits, as is the case for height growth in KLA. The latter probably also indicates for the planted stand VOE a stronger kinship and lesser diversity among the original planting stock (it may have been sourced on a limited amount of mother trees). As BOR was less sampled (only three mother trees) it is more difficult to make assumptions in this respect. The small variability in the quantitative trait height in the putative non-local provenance VOE can be related to the findings of Vranckx et al. (2014b). Based on a molecular genetic analysis (SSR), a stronger relatedness among the pedigrees of typical planted oak stands in the northern part of Belgium compared to the kinship among the mother trees was shown, which was attributed to forest fragmentation, which negatively influences genetic diversity (Vranckx et al., 2012), and is possibly strengthened by the plantation history of the mother trees (Vranckx et al., 2014a).

### Phenological Responses to Water Deficit Followed by Re-watering

In general, leaf senescence follows a highly complex genetic program that is tightly controlled by multiple layers of regulation at the level of transcription, post-transcription, translation and post-translation (Woo et al., 2013). Based on reported results of phenological responses of oak seedlings upon drought treatment (Jensen and Hansen, 2010; Spiess et al., 2012; Kuster et al., 2014), an acceleration of the onset of leaf senescence was expected due to the drought treatment in our experiment, but we observed a clear delay. This can be ascribed to the plentiful re-watering after the drought treatment. It is assumed that enhanced plant growth upon re-watering after a drought period is a compensation for the loss of net primary production due to the preceding water deficit and has been observed in herbaceous plants such as perennial grass (Xu et al., 2009) and in woody species such as oak (Spiess et al., 2012; Turcsán et al., 2016). In perennial grass, the magnitude of this pre-drought stimulation of relative growth rate was found relative to the severity of the drought (Xu et al., 2009). The observed delayed senescence in our experiment can be explained as a compensation time in which plants did not produce an extra shoot but may have taken time to recover before entering the next developmental phenophase of leaf senescence. This recovery may include restoration of the antioxidant metabolism. Similar to other environmental stresses, drought induces a rapid accumulation of reactive oxygen species (ROS), which is reversed upon re-watering (Sofo et al., 2005; Xu et al., 2010). The ROS scavenging mechanisms induced during the recovery phase may interfere with the developmentally regulated autumnal leaf senescence, which includes the degradation of cellular structures in an orderly manner, programmed cell death and the generation of ROS (Woo et al., 2013). Additionally, abscisic acid (ABA) may play a role in the delayed leaf senescence. ABA is a stress hormone well-known to signal drought in plants showing elevated levels during drought periods which are lowered again upon re-watering (Chaves et al., 2003; Xu et al., 2010). As ABA mediates the developmentally regulated autumnal leaf senescence (Woo et al., 2013), drought induced ABA signals may similarly interfere with this process causing the observed delay. This observed compensation time is diminished for seedlings sharing a pot compared to single plants, indicating that a putative competition for resources attenuated the drought and re-watering induced delay in onset of leaf senescence. The higher drought stress experienced by double plants per pot may have weakened their ability of full recover by reducing physiological resilience and may thus have shortened the recovery period and allowed an earlier onset of leaf senescence. It has been shown that severe drought can cause photodamage in Mediterranean tree seedlings that in turn may impair physiological repair and recovery upon re-watering (Benigno et al., 2014). We observed bud burst as the succeeding phenophase following leaf senescence. It has already been shown that a phenological shift in perennials may show an after effect in the subsequent phenophases (Fu et al., 2014). Similarly, we observed a slight delay in bud burst among the drought treated group of plants in the subsequent spring. In this phenological model, the number of plants per pot was not significant, and plant height was significant but independent of the treatment (interaction term with relative weight loss was not significant). Together, our results emphasize the capacity of oaks to resume growth after periods of water deficit. Still, a delayed senescence in autumn will concur with a delayed autumnal hardening and may render shoots vulnerable to early frosts. In addition, a delayed flushing may diminish the competitive capacity of seedlings in relation to competing plants.

## Conclusion

Last decades a strong tendency emerged in the northern part of Belgium to promote natural rejuvenation in a sustainable and nature-oriented forest management. Our results demonstrate that provenance may play an important role in forest regeneration, in the face of the predicted climate change. Although, we found survival rate after a severe water deficit being independent of the provenance, still it was dependent on plant height. A large variability among the seedlings can therefore be advantageous, with smaller plants surviving harsh summer conditions and higher plants profiting from a competitive gain in normal years. Commercial planting stock used in (re)forestations is traditionally graded (Vander Mijnsbrugge et al., 2010), reducing variability and highlighting the value of natural rejuvenation with an option of additional stocking especially in oak stands with a plantation history of unknown origin.

## Author Contributions

KVM, AT, SM, KS, and MS concepted and supervised the here presented experiment, including the organization of the seed collection, while AT, KVM, JM, and ND conducted the measurements and observations on the seedlings. KVM, AT, JM, and ND performed the statistical analysis: AT was responsible for the weight loss and height measurements, AT, JM, and ND for the leaf senescence observations and KV for the bud burst observations. All authors (KVM, AT, JM, NL, SM, MS, KS) contributed substantially to the manuscript preparation.

## Conflict of Interest Statement

The authors declare that the research was conducted in the absence of any commercial or financial relationships that could be construed as a potential conflict of interest.
